# AI readiness for molecular precision medicine supply chains: evidence from Saudi Arabia

**DOI:** 10.3389/fdgth.2026.1910051

**Published:** 2026-08-13

**Authors:** Islam El-Nakib, Karim Soliman

**Affiliations:** 1 Operations and Supply Chain Management Department, College of Business, Effat University, Jeddah, Saudi Arabia; 2 Supply Chain Management Department, College of Business Administration, University of Business and Technology, Jeddah, Saudi Arabia

**Keywords:** artificial intelligence readiness, biobanking, biological sample traceability, biomarker analysis, genomic testing, healthcare supply chain, molecular diagnostics, Saudi Arabia

## Abstract

**Introduction:**

Artificial intelligence (AI) is reshaping molecular precision medicine through biomarker discovery, genomic variant interpretation, molecular imaging, multi-omics analysis, and patient-specific therapeutic support. In Saudi Arabia, genomic medicine and digital health initiatives are expanding, yet limited evidence explains how AI readiness supports molecular diagnostics, genomic testing, biobanking, biological sample traceability, and targeted therapy delivery.

**Methods:**

A structured cross-sectional survey was completed by 350 professionals from hospitals, molecular diagnostic laboratories, genomic testing centers, biomedical laboratories, biobanks, pharmaceutical and biologic suppliers, and healthcare service providers in Saudi Arabia. Data were analyzed using partial least squares structural equation modeling.

**Results:**

AI readiness strengthened molecular data interoperability, biological sample traceability, diagnostic responsiveness, biomarker-guided decision support, and precision medicine service performance. Respondents from more AI-ready organizations reported stronger diagnostic responsiveness and fewer perceived workflow delays.

**Discussion:**

The findings connect AI readiness with molecular diagnostics, genomic services, biomarker analytics, biobanking, and healthcare supply chain coordination, providing evidence to support AI-enabled molecular medicine transformation in Saudi Arabia.

## 1 Introduction

Advances in molecular medicine have transformed healthcare from standardized treatment approaches toward personalized and precision-based care systems. Precision medicine aims to provide tailored interventions according to an individual's genomic profile, molecular characteristics, biomarkers, and disease mechanisms rather than applying generalized therapeutic strategies to broad patient populations (1). The rapid growth of molecular diagnostics, genomic sequencing technologies, biomarker identification methods, and targeted therapies has accelerated this transition and created new opportunities for improving disease prediction, early diagnosis, and patient-specific treatment decisions (2). Molecular medicine has shown significant value in oncology, cardiovascular diseases, neurological disorders, and rare genetic diseases where genetic variability and molecular differences strongly influence treatment effectiveness and patient outcomes (3).

At the molecular level, precision medicine depends on the generation, preservation, interpretation, and clinical use of biological information. Patient samples may pass through pre-analytical, analytical, and post-analytical stages before a clinician receives a molecular result. These stages include biospecimen collection, stabilization, cold-chain transfer, nucleic-acid extraction, sequencing or amplification, biomarker detection, variant classification, molecular reporting, and targeted therapy selection. Weakness in any stage can affect molecular validity, diagnostic turnaround time, and the clinical value of precision medicine. For this reason, AI readiness in molecular healthcare must be studied alongside the workflows that preserve biospecimen quality and connect molecular results with patient-specific decisions (4–6).

Recent developments in artificial intelligence (AI) have further expanded the capabilities of molecular medicine by enabling advanced data processing, predictive modeling, and automated decision support systems (7). AI techniques such as machine learning, deep learning, and neural networks can analyze large volumes of molecular and genomic information that exceed the processing capability of conventional analytical approaches (8). These technologies have demonstrated considerable effectiveness in identifying molecular patterns, predicting biomarker interactions, improving genomic interpretation, and supporting personalized therapeutic recommendations (9). AI applications have also facilitated disease risk prediction, molecular image interpretation, drug discovery, and treatment optimization (4). As healthcare systems increasingly adopt precision medicine approaches, AI has become an important technological mechanism for translating complex biological information into clinically useful knowledge (10).

Despite these technological advances, the successful implementation of molecular precision medicine extends beyond analytical capabilities and computational performance. Precision medicine systems rely on interconnected healthcare networks that coordinate multiple processes involving biological samples, molecular data, laboratory procedures, diagnostic workflows, and targeted therapeutic delivery (11). These systems require collaboration among hospitals, molecular diagnostic laboratories, genomic testing centers, biobanks, pharmaceutical suppliers, biologic manufacturers, and healthcare service providers (12). Biological samples frequently require controlled transportation environments and strict cold-chain conditions to preserve sample integrity and maintain molecular stability (13). Genomic testing procedures also depend on reliable information sharing and coordination among different institutions to support rapid diagnostic interpretation and treatment decisions (14). Consequently, healthcare systems require integrated operational structures that support the movement of biological materials and molecular information throughout the precision medicine process.

From this perspective, molecular precision medicine supply chains represent a critical infrastructure that enables effective precision healthcare delivery (15). Unlike conventional healthcare logistics systems, molecular precision medicine supply chains involve high levels of complexity because they manage biological samples, genomic information, biomarker testing procedures, personalized therapies, and patient-specific interventions (16). Biological samples such as tissue specimens, blood samples, and genetic material frequently move across multiple laboratories and healthcare institutions before clinical interpretation and treatment decisions occur (17). Failures in sample handling, delays in laboratory processing, poor data interoperability, or weak coordination among healthcare organizations may negatively influence diagnostic quality and patient outcomes (17). Therefore, supply chains within molecular medicine should not be viewed solely as operational systems but rather as integrated healthcare networks that support molecular research translation and clinical applications.

Artificial intelligence readiness has recently emerged as an important factor influencing healthcare digital transformation and technological adoption (17). AI readiness reflects the extent to which organizations possess the technological infrastructure, organizational capabilities, human resources, regulatory support, and data management capabilities required to implement AI technologies effectively (18). Organizations with higher AI readiness levels are more capable of integrating intelligent systems into operational and clinical environments (19). Existing studies have examined AI readiness in healthcare settings, digital transformation contexts, and organizational technology adoption environments (8). However, previous research has largely focused on operational efficiency, technology implementation, and general healthcare applications without examining molecular medicine systems specifically (19).

This limitation becomes particularly important in emerging healthcare environments where digital transformation strategies increasingly support advanced healthcare technologies. Saudi Arabia has recently undertaken substantial healthcare reforms through Vision 2030 initiatives aimed at strengthening healthcare quality, digital health systems, and biomedical innovation (20). The country has invested in healthcare infrastructure, AI technologies, genomic initiatives, and digital healthcare services to improve healthcare accessibility and support precision medicine development (7). National programs such as the Saudi Human Genome Program have further accelerated interest in genomic medicine and personalized healthcare approaches (21). The integration of AI technologies into molecular diagnostics and precision healthcare systems has therefore become increasingly relevant within the Saudi healthcare environment.

Recent Saudi and MENA supply chain studies further show that regional healthcare and logistics transformation depends on organizational readiness, human capability, integration, and governance capacity (22–25). These findings support the present study's emphasis on AI readiness as a molecular-healthcare network capability rather than a narrow technology adoption issue.

Despite these developments, limited evidence explains how healthcare organizations develop AI readiness capabilities that support molecular precision medicine systems and their associated supply chain structures (26). Existing studies have generally examined AI implementation from technological or clinical perspectives while paying limited attention to organizational readiness, healthcare network integration, and molecular service workflows (27). Similarly, research investigating healthcare supply chains often focuses on operational efficiency, inventory management, or healthcare logistics rather than molecular diagnostics, genomic testing coordination, and biomarker-based decision systems (28). This creates an important gap in understanding the mechanisms through which AI readiness influences molecular precision medicine operations and healthcare service performance.

To address this gap, the present study investigates the determinants and outcomes of AI readiness within molecular precision medicine supply chains in Saudi Arabia. Drawing on the Technology-Organization-Environment framework and healthcare network theory, the study examines how technological capability, organizational readiness, data governance, regulatory support, and supply chain integration influence AI readiness among biomedical, diagnostic, genomic, and healthcare service providers. The study further examines how AI readiness contributes to molecular data interoperability, biological sample traceability, genomic testing responsiveness, biomarker-guided decision support, and precision medicine service performance.

The novelty of this study lies in shifting AI readiness research from general digital health adoption to the specific operational and organizational conditions required for molecular precision medicine supply chains. Prior studies have examined AI in clinical medicine, genomics, diagnostics, or healthcare logistics separately. However, limited empirical evidence explains how AI readiness supports the coordinated movement of biospecimens, genomic information, biomarker outputs, biobank-linked sample metadata, diagnostic reporting, and targeted therapy decisions across healthcare networks. This study addresses that gap by integrating the TOE framework with Healthcare Network Theory and empirically testing AI readiness as a network-level capability within Saudi Arabia's molecular precision medicine ecosystem.

This study provides several contributions. First, it extends AI readiness research into molecular medicine and precision healthcare environments. Second, it integrates supply chain coordination with molecular healthcare systems and positions supply chains as enabling infrastructures for precision medicine delivery rather than traditional logistics mechanisms. Third, it provides empirical guidance for healthcare managers and policymakers seeking to strengthen AI-enabled molecular healthcare systems under Saudi Arabia's healthcare transformation agenda. Finally, the study contributes to the growing literature on AI applications in molecular medicine by connecting technological readiness with molecular healthcare outcomes and patient-centered treatment systems.

**Figure 1:**
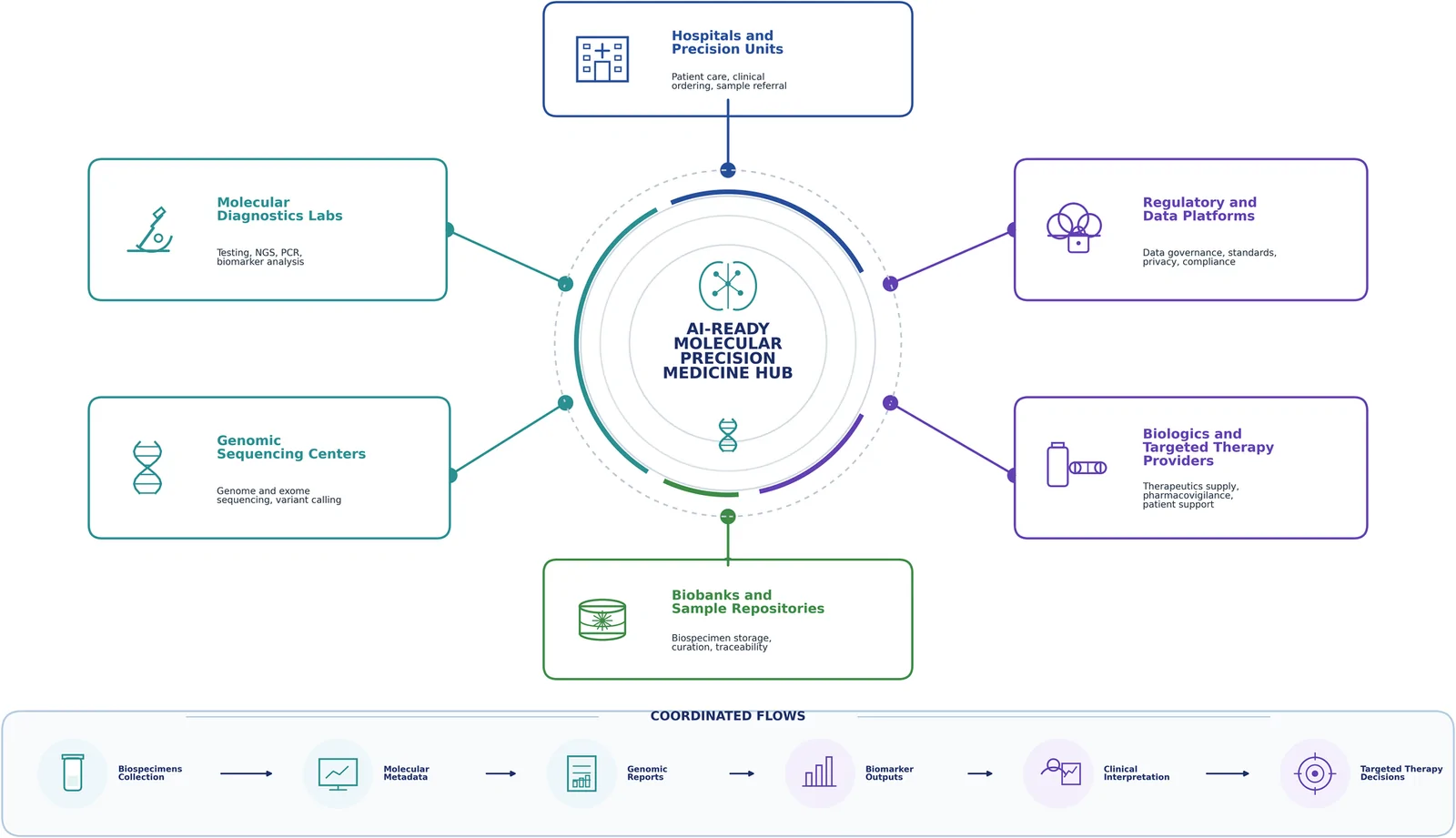
Saudi molecular precision medicine ecosystem for AI-ready service delivery.

The Saudi context requires an ecosystem view because AI-ready molecular medicine depends on coordination across care providers, laboratories, genomic centers, biobanks, regulatory platforms, and targeted therapy providers (29–35). Figure 1 illustrates the Saudi molecular precision medicine ecosystem for AI-ready service delivery.

Figure 1 frames integration as infrastructure for molecular research translation. It shows how biospecimens, molecular metadata, genomic reports, biomarker outputs, clinical interpretation, and targeted therapy decisions move across the Saudi precision medicine ecosystem (5, 6).

## 2 Literature review

### 2.1 Artificial intelligence in molecular medicine

Artificial intelligence has become one of the most important technological developments influencing molecular medicine and precision healthcare systems. AI refers to computational approaches that allow systems to perform analytical tasks that traditionally require human intelligence, including pattern recognition, prediction, learning, and decision support (36). The rapid expansion of machine learning, deep learning, and neural network approaches has significantly changed the way biological and clinical information is processed and interpreted (37). Traditional analytical approaches often encounter limitations when handling large volumes of heterogeneous molecular data due to computational complexity and the multidimensional nature of biological systems (38). Molecular medicine increasingly generates large-scale datasets from genomic sequencing, proteomics, transcriptomics, metabolomics, and molecular imaging platforms that require advanced analytical capabilities (39).

AI technologies have demonstrated substantial potential for improving the interpretation of biological information and supporting precision medicine applications. Machine learning algorithms can identify hidden patterns and relationships within molecular datasets that may remain undetected using conventional statistical techniques (40). Deep learning approaches have shown particular effectiveness in recognizing nonlinear relationships among genes, proteins, biomarkers, and disease pathways (41). Such capabilities have contributed to improved disease prediction and individualized treatment planning.

Recent studies indicate that AI applications in molecular medicine have expanded substantially across several areas. One important application involves disease risk prediction. AI systems can analyze molecular signatures and genomic patterns to identify patients at higher risk of disease development (41). Researchers have reported successful use of machine learning models for predicting cancer progression, cardiovascular diseases, diabetes, and neurodegenerative disorders (42). AI systems have also been used for molecular imaging interpretation, allowing automated analysis of pathology images and radiological examinations with improved speed and accuracy (43).

Drug discovery represents another important area where AI contributes to molecular medicine. Conventional drug discovery processes frequently require extensive time and resources due to the large number of compounds and biological interactions involved (44). AI models can accelerate candidate identification through predictive algorithms that analyze molecular structures, biological interactions, and therapeutic outcomes (45). Deep learning approaches have also been used to support drug repurposing efforts and targeted therapy development (46).

Despite substantial progress, several barriers limit effective AI implementation within molecular healthcare systems. Healthcare organizations frequently encounter challenges related to technological infrastructure, data quality, workforce capability, and regulatory compliance (47). The integration of AI into molecular healthcare systems therefore depends not only on computational performance but also on organizational readiness and operational capabilities. Consequently, AI readiness has emerged as an important concept for understanding how healthcare institutions develop capabilities required for successful implementation (5).

### 2.2 Genomics and biomarker analytics

Genomics and biomarker research represent fundamental components of precision medicine because they provide insights into biological variability among individuals and support personalized treatment decisions (6). Genomics refers to the study of genetic structures, functions, interactions, and variations within organisms, while biomarkers are measurable biological characteristics that indicate physiological conditions, disease states, or therapeutic responses (48). Advances in sequencing technologies have significantly improved the accessibility and affordability of genomic analysis, resulting in substantial growth in molecular healthcare applications (49).

Genomic technologies generate large amounts of biological information that can improve disease detection and treatment strategies. Next-generation sequencing technologies have enabled comprehensive analysis of genetic variations associated with disease development and progression (50). Such technologies allow researchers and healthcare practitioners to identify genetic mutations and biological mechanisms associated with specific diseases (51). Precision medicine initiatives increasingly rely on genomic testing to support individualized healthcare interventions.

Biomarkers play a critical role in precision healthcare because they provide measurable indicators that can support diagnosis, prognosis, and treatment selection (52). Biomarkers may include proteins, genes, metabolites, molecular signatures, and physiological indicators associated with biological processes (29). Biomarker applications have expanded substantially in cancer treatment, where molecular markers frequently support treatment selection and therapeutic monitoring (20). Biomarker-based approaches also support disease screening and risk assessment.

AI has increasingly become integrated into genomic and biomarker analytics because of the complexity and scale of molecular datasets. AI systems can identify biomarker interactions and molecular patterns that are difficult to detect through conventional analytical methods (53). Machine learning algorithms have demonstrated strong capabilities for analyzing genomic sequences and predicting disease outcomes (54). AI-based biomarker analytics also support personalized treatment recommendations and disease monitoring (53).

However, genomic and biomarker applications generate several operational and organizational challenges. Healthcare systems must coordinate large volumes of sensitive patient information and biological data while ensuring privacy, security, and interoperability (53). Effective genomic testing processes also depend on coordination among laboratories, healthcare providers, and diagnostic centers (54). Consequently, healthcare systems require integrated infrastructures capable of supporting data exchange and collaborative workflows.

**Figure 2:**
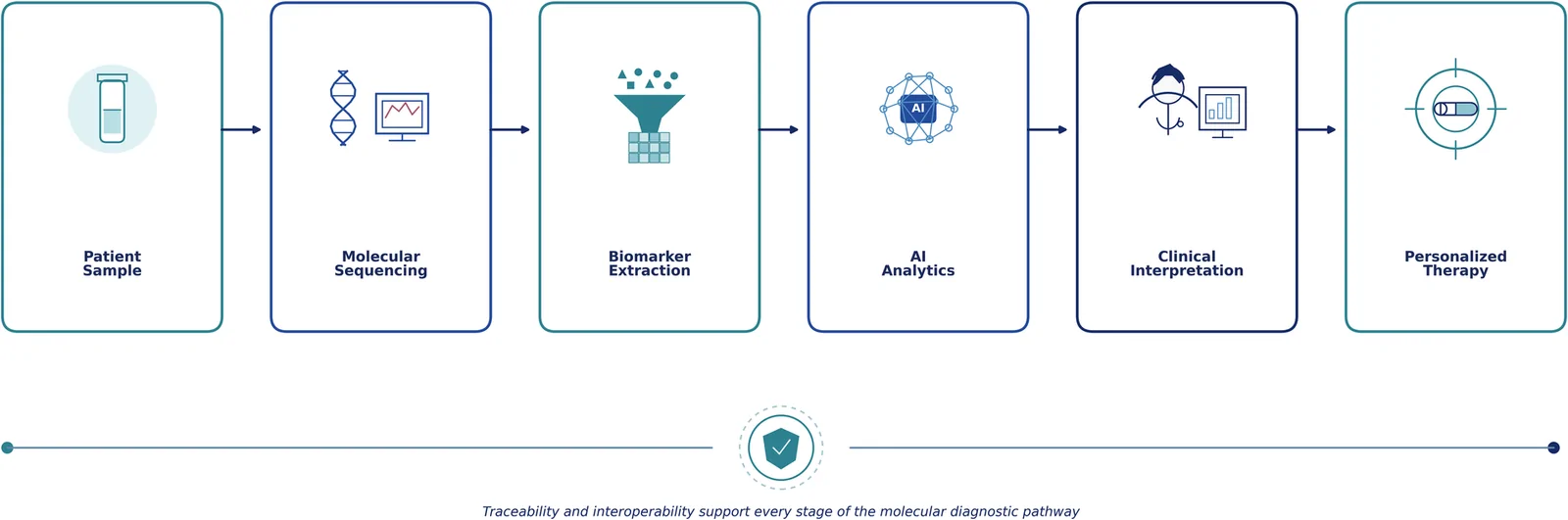
AI workflow for biomarker and genomic analytics.

Genomic and biomarker analytics require a staged pathway in which biological samples are converted into clinically useful molecular evidence. Figure 2 illustrates how AI supports the movement from sequencing and biomarker extraction to clinical interpretation and personalized therapy (11, 14, 40).

Figure 2 shows that AI readiness is relevant across the molecular diagnostic pathway. Its value includes prediction, traceability, interoperability, interpretation quality, and patient-specific treatment support (18, 19, 41–43).

### 2.3 Molecular diagnostics

Molecular diagnostics refers to laboratory techniques that analyze biological markers at molecular levels to identify diseases, genetic conditions, and treatment responses (53). Molecular diagnostic technologies have significantly improved healthcare capabilities by enabling earlier disease detection and more accurate diagnosis (53). Such approaches analyze DNA, RNA, proteins, and other molecular components associated with disease mechanisms (53).

Several molecular diagnostic methods are commonly used in healthcare practice. Polymerase chain reaction techniques allow amplification and analysis of genetic material for disease detection (55). Next-generation sequencing technologies support large-scale genomic analysis and mutation identification (56). Other molecular diagnostic approaches include microarray analysis and biomarker testing systems (57).

The integration of AI technologies has further strengthened molecular diagnostic systems. AI-based diagnostic tools can analyze molecular patterns and assist healthcare professionals in identifying disease characteristics (58). Machine learning models have demonstrated strong predictive capabilities in cancer diagnostics, infectious disease detection, and genomic mutation analysis (59).

Nevertheless, molecular diagnostic systems involve complex operational processes requiring coordination among multiple healthcare entities (58). Biological samples frequently move across laboratories and healthcare institutions before diagnostic interpretation occurs (60). Delays in testing procedures or failures in sample management may reduce diagnostic effectiveness (61). Therefore, healthcare systems require reliable infrastructures capable of supporting efficient diagnostic workflows.

### 2.4 Biospecimen integrity, biobanking, and molecular research translation

Molecular precision medicine depends on the integrity of biospecimens and the reliability of sample-linked data. Biological samples often move from clinical collection sites to processing laboratories, sequencing facilities, biobanks, or external reference laboratories. During these movements, sample identity, temperature stability, chain of custody, and metadata accuracy must be preserved. Pre-analytical error can weaken molecular validity because poor handling may alter DNA, RNA, protein, or metabolite quality before testing occurs (50–52).

Biobanks also play a central role in molecular research translation because they connect biospecimens with clinical, genomic, and phenotypic information. AI-ready molecular systems require interoperable repositories in which sample metadata, consent status, assay outputs, and clinical annotations can be linked securely. This requirement connects molecular science with organizational readiness and healthcare network coordination. Without traceable samples and reliable data governance, AI systems may process incomplete or inconsistent molecular information, limiting their clinical value (5, 6, 48).

Targeted therapy workflows further strengthen the need for molecular integration. Biomarker-guided treatment decisions require timely movement from molecular testing to clinical interpretation and therapeutic selection. When genomic results, biomarker panels, and sample histories are fragmented across institutions, personalized therapy decisions may be delayed. AI readiness therefore becomes a network-level capability that supports molecular research translation from biospecimen to result, from result to interpretation, and from interpretation to patient-specific treatment planning.

**Figure 3:**
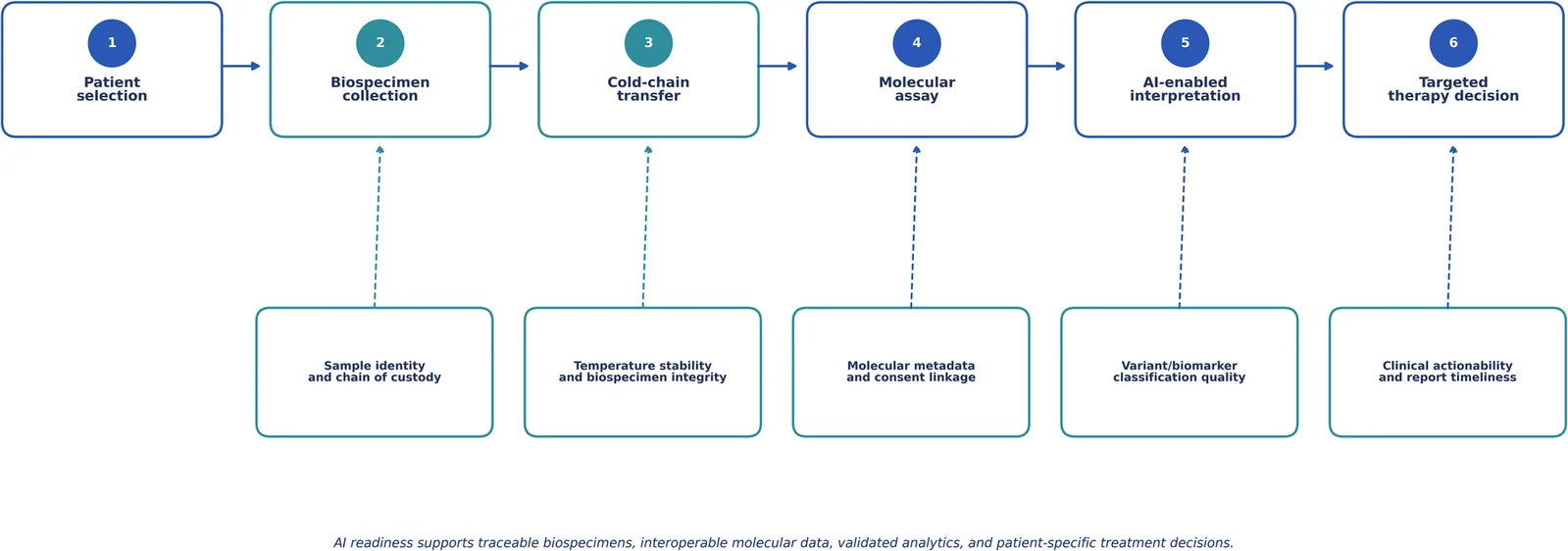
AI-enabled molecular precision medicine workflow from biospecimen collection to targeted therapy decision.

Molecular research translation depends on reliable biospecimen handling before clinical interpretation can occur. Figure 3 presents the biospecimen-to-therapy pathway and highlights control points that support molecular validity (50–52, 62).

Figure 3 emphasizes that biospecimen quality, cold-chain transfer, metadata linkage, variant classification, and report timeliness are core conditions for valid molecular interpretation and targeted therapy decisions (28, 50–52).

### 2.5 Precision medicine supply chains

Supply chain systems play an important role in supporting precision medicine because healthcare delivery increasingly depends on coordinated movement of biological samples, molecular data, diagnostic outputs, and personalized therapies (63). Traditional healthcare supply chains largely focus on inventory management and physical product distribution (64). However, precision medicine systems involve additional complexity because they support individualized treatment pathways and molecular testing procedures (64).

Precision medicine supply chains involve multiple participants including hospitals, laboratories, genomic centers, biobanks, pharmaceutical providers, and healthcare institutions (65). Biological samples frequently move between collection sites and analytical facilities, while molecular data require secure and efficient transmission (30). Such processes require coordination among multiple stakeholders to ensure reliability and timeliness.

Cold-chain management also becomes important in molecular precision medicine systems because biological materials frequently require controlled environmental conditions (31). Failures in temperature control or transportation processes may compromise sample quality and reduce diagnostic validity (32). Sample traceability therefore becomes critical for maintaining data integrity and patient safety.

AI technologies have increasingly supported precision medicine supply chain systems through predictive analytics and decision-support capabilities (32). Intelligent systems can optimize resource allocation and improve coordination across healthcare networks (29). However, healthcare organizations require sufficient technological and organizational readiness before obtaining these benefits (33).

**Figure 4:**
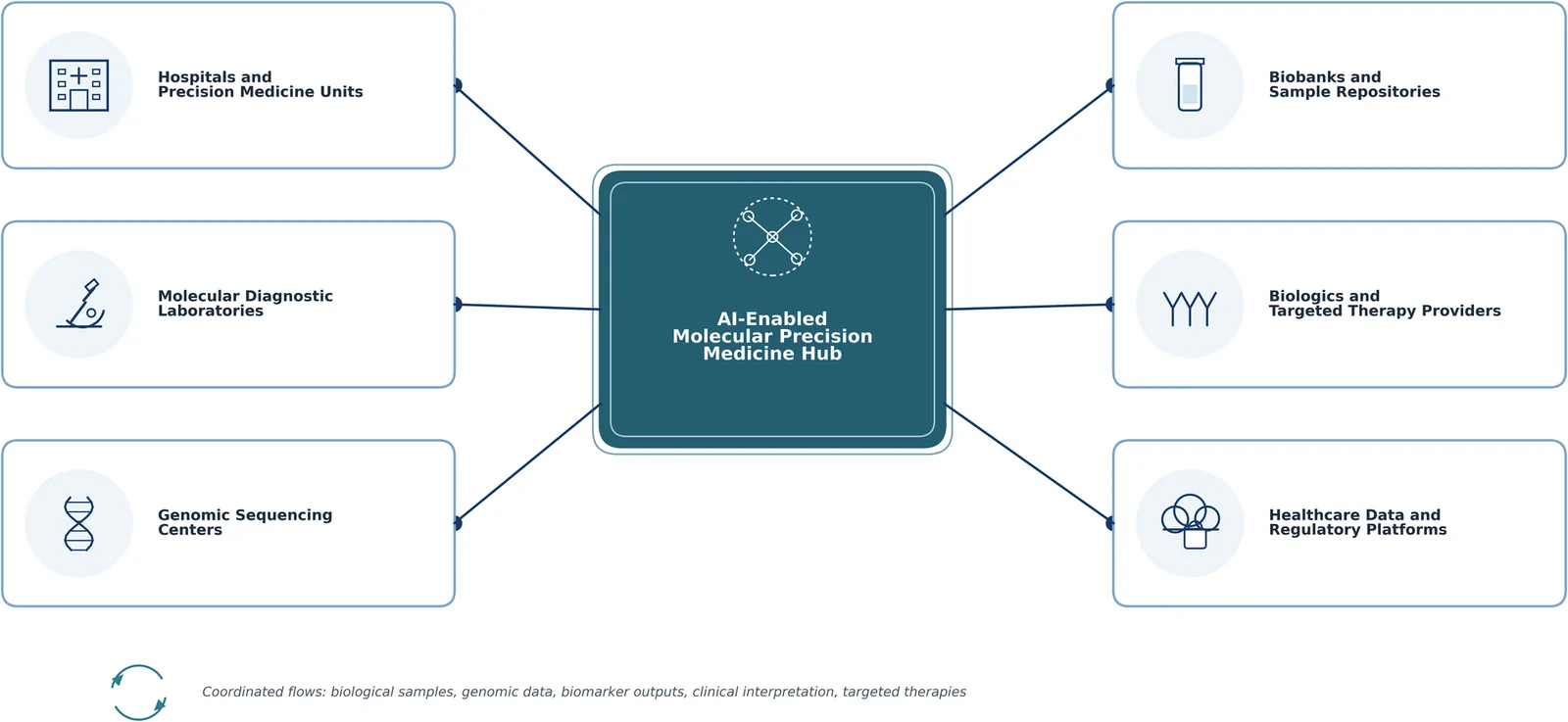
Molecular precision medicine network architecture.

Precision medicine supply chains operate as molecular healthcare networks rather than linear delivery systems. Figure 4 shows the institutional actors involved in AI-enabled molecular precision medicine and the coordinated flows that connect them (47, 53, 54).

Figure 4 supports the use of Healthcare Network Theory in this study. Molecular precision medicine outcomes depend on how well hospitals, laboratories, genomic centers, biobanks, data platforms, and therapy providers exchange samples, data, reports, and treatment information (47, 66).

### 2.6 Saudi vision 2030 and digital health transformation

Saudi Arabia has implemented major healthcare reforms under Vision 2030 to improve healthcare quality and strengthen healthcare accessibility (34). Digital transformation initiatives have emphasized technological innovation, AI implementation, and healthcare modernization (35). Healthcare institutions have increasingly adopted digital platforms and advanced analytical technologies (31).

The Saudi Human Genome Program represents one of the country's major initiatives supporting genomic medicine development (31). The program aims to identify genetic characteristics associated with diseases within the Saudi population and improve personalized healthcare interventions (31). Precision medicine initiatives have also received increasing attention within national healthcare policies.

AI technologies have become an important component of healthcare transformation strategies within Saudi Arabia (31). Government initiatives have supported AI implementation across healthcare services including disease prediction and clinical decision support systems (31). However, organizational readiness and healthcare integration challenges continue to affect implementation efforts (46).

### 2.7 Research gaps

Although previous studies have examined AI implementation, molecular diagnostics, genomics, and healthcare digital transformation separately, several important research gaps remain. First, existing studies frequently focus on technological capabilities without examining organizational and environmental readiness factors that influence AI implementation in molecular healthcare environments (48).

Second, research investigating precision medicine frequently emphasizes clinical outcomes while paying limited attention to operational infrastructures and healthcare coordination mechanisms (48). Molecular healthcare systems depend heavily on integrated workflows involving laboratories, genomic centers, biobanks, and healthcare providers. Limited studies examine these systems from a healthcare network perspective (47).

Third, healthcare supply chain studies frequently focus on conventional logistics functions such as inventory management and service efficiency rather than molecular diagnostics and genomic workflows (19). Limited evidence explains how AI readiness contributes to molecular data interoperability, biological sample traceability, and biomarker-guided decision support (8).

Fourth, limited empirical evidence exists regarding AI readiness within Saudi molecular healthcare systems despite increasing national investment in healthcare digital transformation (4). Existing studies have not comprehensively examined the interactions among technological capability, organizational readiness, supply chain integration, and molecular healthcare outcomes. Recent regional studies confirm that MENA supply chain research needs stronger theory-driven, context-sensitive models and that Saudi healthcare supply chains face capability and coordination barriers that affect digital transformation (22, 23, 25).

To address these limitations, the present study develops an integrated framework based on the Technology-Organization-Environment model and healthcare network theory to examine AI readiness in molecular precision medicine supply chains and its impact on molecular healthcare performance outcomes. Table 1 presents the literature synthesis matrix.

**Table 1:** Literature synthesis matrix.

Stream	Representative focus	Relevance to current study	Identified gap
AI in molecular medicine	Machine learning, deep learning, molecular pattern recognition	Explains how AI converts complex molecular data into clinical insight	Limited attention to organizational readiness and supply-chain coordination
Genomics and biomarkers	Sequencing, genomic variants, biomarker discovery	Provides molecular basis for personalized diagnosis and treatment	Limited integration with healthcare workflow and traceability issues
Molecular diagnostics	PCR, NGS, biomarker testing, laboratory workflows	Connects molecular testing with diagnostic responsiveness	Operational coordination and AI readiness remain underdeveloped
Precision medicine supply chains	Biological samples, cold chains, laboratory networks	Frames supply chains as enabling molecular medicine infrastructure	Often treated as logistics efficiency rather than molecular research translation
Saudi digital health	Vision 2030, Saudi genome initiatives, AI strategy	Provides national context for empirical study	Saudi molecular AI readiness evidence remains limited

## 3 Theoretical background and hypotheses development

### 3.1 Theoretical background

This study adopts the Technology-Organization-Environment framework and Healthcare Network Theory to explain the determinants and outcomes of AI readiness within molecular precision medicine supply chains. The integration of these theoretical perspectives provides a comprehensive understanding of how technological capabilities, organizational conditions, and environmental influences shape AI adoption while considering the interconnected nature of molecular healthcare systems. Tables 2–4 summarize the hypotheses, target respondent categories and expertise areas, and proposed constructs and measurement indicators, respectively.

**Table 2:** Hypotheses summary.

Hypothesis	Proposed relationship	Direction
H1	Technological capability positively influences AI readiness within molecular precision medicine supply chains.	Positive
H2	Organizational readiness positively influences AI readiness within molecular precision medicine supply chains.	Positive
H3	Data governance positively influences AI readiness within molecular precision medicine supply chains.	Positive
H4	Regulatory support positively influences AI readiness within molecular precision medicine supply chains.	Positive
H5	Supply chain integration positively influences AI readiness within molecular precision medicine supply chains.	Positive
H6	AI readiness positively influences molecular data interoperability.	Positive
H7	AI readiness positively influences biological sample traceability.	Positive
H8	AI readiness positively influences diagnostic responsiveness.	Positive
H9	AI readiness positively influences biomarker-guided decision support.	Positive
H10	AI readiness positively influences precision medicine service performance.	Positive

**Table 3:** Target respondent categories and expertise areas.

Respondent category	Role	Targeted sample coverage
Hospital administrators	Healthcare management	15%
Molecular diagnostic specialists	Laboratory operations	20%
Genomic testing specialists	Genomic analysis	15%
Biomedical laboratory personnel	Molecular testing	15%
Supply chain managers	Logistics coordination	10%
Precision medicine specialists	Clinical applications	15%
AI technology specialists	AI implementation	10%

**Table 4:** Proposed constructs and measurement indicators.

Construct	Code	Sample measurement item
Technological capability	TC	Our organization possesses sufficient technological infrastructure for AI implementation.
Organizational readiness	OR	Top management supports AI implementation initiatives.
Data governance	DG	Molecular data are managed through secure governance mechanisms.
Regulatory support	RS	Existing regulations facilitate AI implementation.
Supply chain integration	SCI	Our organization exchanges molecular information effectively with external healthcare partners.
AI readiness	AIR	Our organization is prepared to implement AI systems in molecular precision medicine workflows.
Molecular interoperability	MI	Molecular information can be exchanged across systems and institutions.
Sample traceability	ST	Biological samples are traceable throughout testing and storage workflows.
Diagnostic responsiveness	DR	Molecular diagnostic activities are completed rapidly and reliably.
Biomarker decision support	BDS	Biomarker information supports patient-specific treatment decisions.
Service performance	SP	Precision medicine services operate effectively and support patient-centered outcomes.

The TOE framework, developed by Tornatzky and Fleischer (55), explains organizational technology adoption through three major dimensions: technological context, organizational context, and environmental context. The technological dimension includes the availability and characteristics of technologies necessary for adoption. The organizational dimension addresses internal resources, infrastructure, management support, and organizational capabilities. The environmental dimension includes external factors such as regulations, institutional support, competition, and collaboration networks (56). Previous studies have applied TOE to explain digital transformation, healthcare technology implementation, and AI adoption because it captures both technological and contextual determinants affecting implementation success (58).

In molecular precision medicine environments, the technological context reflects factors such as AI infrastructure, data processing capability, interoperability systems, and molecular information management platforms. Molecular medicine systems depend on large-scale genomic and biomarker datasets requiring advanced analytical systems and high computational capacity (15). Healthcare organizations with stronger technological capability may therefore exhibit greater readiness for AI implementation.

The organizational context refers to institutional capabilities required to support AI integration. These include workforce competence, leadership support, organizational culture, and resource availability (67). Precision medicine environments require specialized expertise in genomics, molecular diagnostics, and AI-supported healthcare systems. Organizations with greater readiness may therefore demonstrate stronger AI implementation capability.

The environmental context represents external conditions affecting organizational behavior. In molecular medicine systems, these conditions include healthcare regulations, governmental support, industry collaboration, and data governance structures (5). Regulatory mechanisms and institutional support can influence AI implementation because molecular healthcare systems frequently involve highly sensitive patient information and biological data (68).

Although TOE effectively explains technology adoption conditions, it does not explicitly address the inter-organizational relationships characterizing molecular healthcare systems. Consequently, Healthcare Network Theory complements the TOE framework by explaining interactions among multiple healthcare entities involved in precision medicine ecosystems.

Healthcare Network Theory proposes that healthcare performance depends on coordination, information exchange, and collaborative relationships among organizations (66). Molecular precision medicine systems involve interconnected networks of hospitals, molecular laboratories, genomic testing centers, biobanks, biologic suppliers, and healthcare providers (47). These entities continuously exchange biological samples, molecular data, diagnostic information, and treatment recommendations. Effective network integration therefore supports operational coordination and healthcare performance.

Combining TOE and Healthcare Network Theory allows this study to explain both organizational AI readiness and healthcare network interactions within molecular precision medicine supply chains.

**Figure 5:**
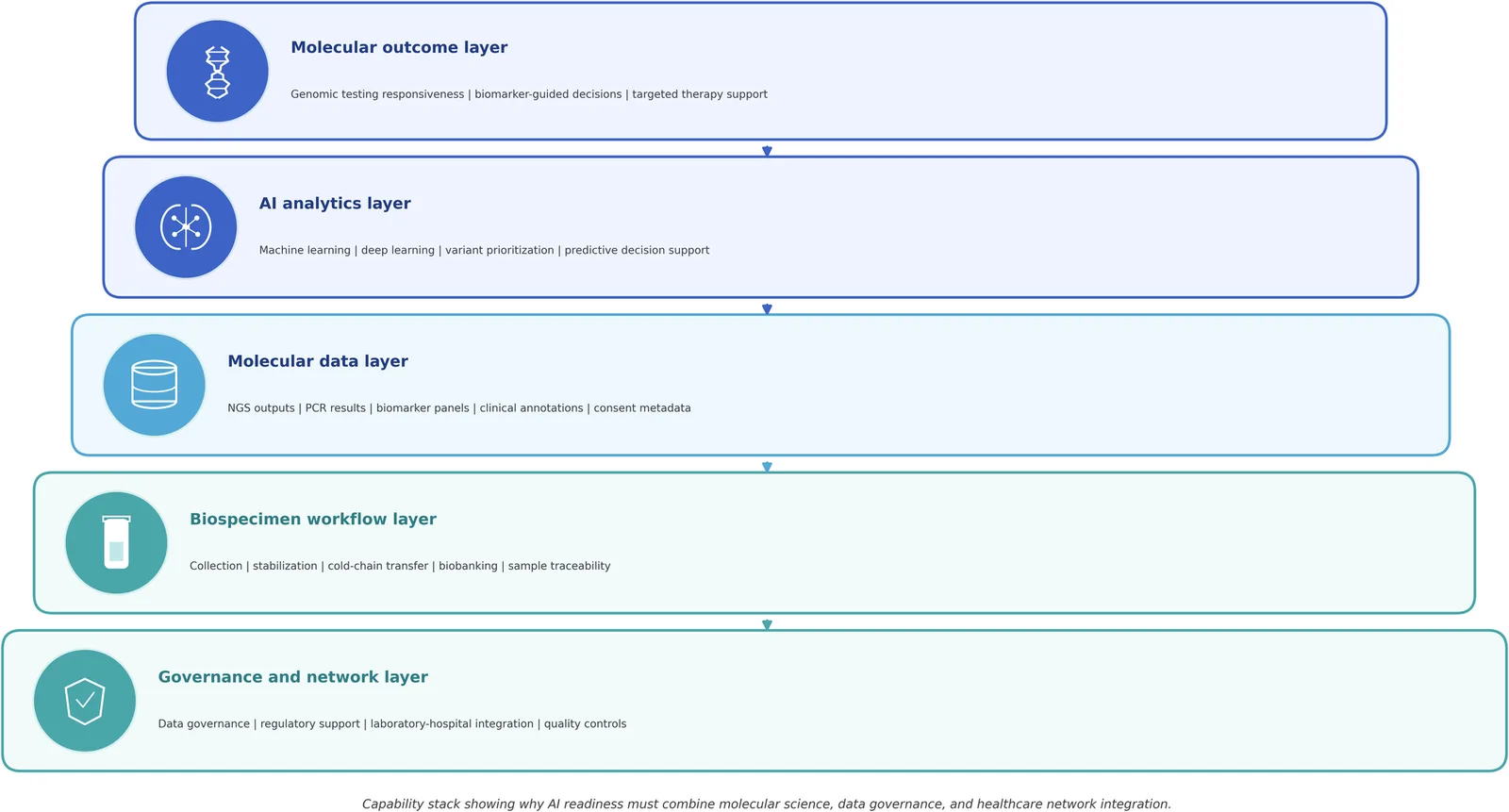
AI readiness capability stack for molecular precision medicine supply chains.

The combined TOE and Healthcare Network Theory logic can be understood as a layered capability stack. Figure 5 shows how governance and network coordination support biospecimen workflows, molecular data quality, AI analytics, and molecular precision medicine outcomes (55, 56, 66, 69).

Figure 5 explains why AI readiness cannot be reduced to software availability. In molecular precision medicine, readiness emerges from the alignment of governance, biospecimen control, molecular data quality, analytical capability, and outcome-focused clinical use (5, 6, 67, 69).

### 3.2 Hypotheses development

#### 3.2.1 H1. Technological capability

Technological capability refers to the availability of infrastructure, analytical systems, computational resources, and technological platforms necessary for AI implementation (7). Molecular precision medicine systems require substantial computational capabilities because genomic sequencing, biomarker analysis, and molecular diagnostics generate large-scale datasets (39). Organizations with advanced technological resources are more likely to possess the infrastructure needed to implement AI applications effectively.

H1: Technological capability positively influences AI readiness within molecular precision medicine supply chains.

#### 3.2.2 H2. Organizational readiness

Organizational readiness refers to the extent to which institutions possess resources, managerial support, skilled personnel, and organizational commitment necessary for technology implementation (67). Molecular healthcare systems require multidisciplinary expertise involving genomics, molecular biology, healthcare operations, and AI systems (47). Organizations with stronger internal capabilities demonstrate greater readiness for AI adoption.

H2: Organizational readiness positively influences AI readiness within molecular precision medicine supply chains.

#### 3.2.3 H3. Data governance

AI systems depend heavily on high-quality and secure data environments. Data governance refers to policies and practices that ensure data integrity, privacy, security, and accessibility (69). Molecular precision medicine environments involve highly sensitive patient information and genomic datasets requiring robust governance systems (69). Organizations with stronger governance systems demonstrate greater readiness for AI implementation.

H3: Data governance positively influences AI readiness within molecular precision medicine supply chains.

#### 3.2.4 H4. Regulatory support

Regulatory support includes policies, legal frameworks, and institutional mechanisms facilitating technological implementation (68). Molecular healthcare systems involve complex ethical and regulatory considerations related to genomic information and biological data (68). Supportive regulations may reduce implementation uncertainty and strengthen organizational confidence.

H4: Regulatory support positively influences AI readiness within molecular precision medicine supply chains.

#### 3.2.5 H5. Supply chain integration

Supply chain integration reflects coordination and information exchange among healthcare entities involved in molecular medicine processes (53). Effective integration supports biological sample movement, molecular information exchange, and diagnostic coordination (53). Organizations with stronger integration demonstrate higher AI readiness levels.

H5: Supply chain integration positively influences AI readiness within molecular precision medicine supply chains.

#### 3.2.6 H6. AI readiness

Molecular precision medicine depends on efficient integration and exchange of molecular information across healthcare systems (6). Organizations with stronger AI readiness demonstrate improved interoperability capabilities.

H6: AI readiness positively influences molecular data interoperability.

#### 3.2.7 H7. AI readiness

Biological sample traceability ensures monitoring and tracking of biological materials across healthcare systems (62). AI technologies can improve monitoring, reduce errors, and improve the reliability of sample-linked molecular data.

H7: AI readiness positively influences biological sample traceability.

#### 3.2.8 H8. AI readiness

Diagnostic responsiveness refers to the speed and effectiveness of molecular testing processes (51). AI systems can improve analytical efficiency and accelerate diagnostic interpretation.

H8: AI readiness positively influences diagnostic responsiveness.

#### 3.2.9 H9. AI readiness

Biomarker-driven clinical decisions require integration of molecular information and predictive analytics (38). AI systems can identify molecular patterns and support individualized treatment recommendations.

H9: AI readiness positively influences biomarker-guided decision support.

#### 3.2.10 H10. AI readiness

Precision medicine service performance reflects healthcare outcomes associated with personalized healthcare delivery, patient-specific treatment support, and service effectiveness (27). Organizations with stronger AI readiness achieve improved healthcare performance.

H10: AI readiness positively influences precision medicine service performance.

## 4 Conceptual framework

The conceptual framework positions AI readiness as a central organizational capability linking TOE and healthcare network inputs with molecular precision medicine outcomes. The left side of the model captures technological capability, organizational readiness, data governance, regulatory support, and supply chain integration. The right side captures outcomes that are specific to molecular medicine, including molecular data interoperability, biological sample traceability, diagnostic responsiveness, biomarker-guided decision support, and precision medicine service performance.

**Figure 6:**
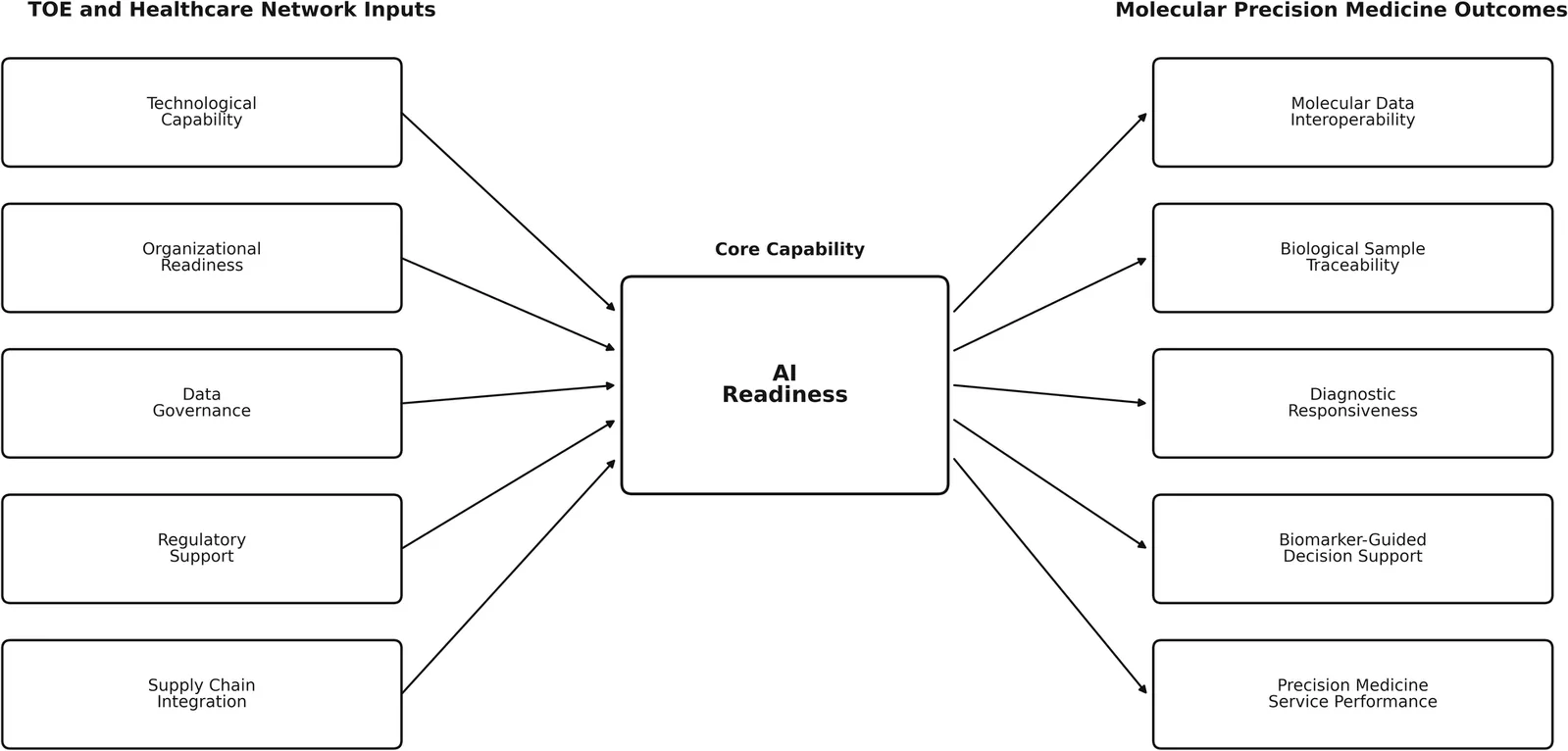
Conceptual framework of AI readiness in molecular precision medicine supply chains.

The proposed framework integrates TOE-based readiness conditions with healthcare network coordination. Figure 6 shows how technological, organizational, governance, regulatory, and integration inputs shape AI readiness and molecular precision medicine outcomes (55, 56, 66).

Figure 6 positions AI readiness as the central capability through which institutional and network conditions affect molecular outcomes. The model links readiness to interoperability, sample traceability, diagnostic responsiveness, biomarker-guided decision support, and precision medicine service performance (18, 19, 53).

## 5 Materials and methods

### 5.1 Research design and study context

This study adopted a quantitative cross-sectional research design to examine the determinants and outcomes of artificial intelligence readiness within molecular precision medicine supply chains in Saudi Arabia. The quantitative approach was appropriate because the study aimed to test hypothesized relationships among multiple latent constructs and evaluate the strength and significance of direct relationships within the proposed theoretical framework (60).

The study integrated the Technology-Organization-Environment framework and Healthcare Network Theory to examine how technological capability, organizational readiness, data governance, regulatory support, and supply chain integration influence AI readiness and molecular precision medicine service outcomes. The TOE framework is suitable for explaining organizational technology adoption through technological, organizational, and environmental conditions (55, 56), while Healthcare Network Theory explains how interorganizational coordination and information exchange influence healthcare system performance (66). Given the complexity of the proposed model and the presence of multiple latent constructs, partial least squares structural equation modeling was selected as the analytical method (60, 61).

The empirical context was Saudi Arabia's molecular precision medicine ecosystem. The study focused on organizations involved in healthcare digital transformation, molecular diagnostics, genomic testing, biobanking, biological sample traceability, biomarker analytics, targeted therapy coordination, and AI-enabled healthcare services. The organizational setting included hospitals and medical centers, precision medicine units, molecular diagnostic laboratories, genomic sequencing centers, biomedical laboratories, biobanks, pharmaceutical and biologics providers, healthcare data platforms, and related healthcare service institutions. This context is relevant because Saudi healthcare transformation initiatives increasingly emphasize digital health, genomics, AI adoption, and biomedical innovation (29, 30).

The survey was designed to capture molecular workflow capability rather than general logistics capability. Therefore, the questionnaire items referred to genomic testing responsiveness, molecular diagnostic turnaround time, biomarker-panel reporting, biobank-linked sample traceability, sample-linked data integrity, molecular data interoperability, and support for targeted therapy decisions. This framing ensured that the empirical model evaluated molecular precision medicine supply chain performance rather than broad healthcare logistics efficiency. The focus on biospecimen integrity, molecular data exchange, genomic reporting, and biomarker-guided decision support is consistent with precision medicine and molecular healthcare workflow literature (5, 6).

### 5.2 Sampling procedure, eligibility criteria, and data screening

A purposive sampling approach was used because the study required respondents with specialized knowledge of molecular precision medicine, healthcare operations, artificial intelligence, and supply chain coordination. Purposive sampling is appropriate when participants must possess domain-specific expertise relevant to the research question (70). The sampling frame included hospitals and precision medicine units, molecular diagnostic laboratories, genomic sequencing centers, biomedical laboratories, biobanks, biologics and targeted therapy providers, healthcare data platforms, and related healthcare service organizations in Saudi Arabia.

Respondents were eligible to participate if they worked in healthcare, molecular diagnostics, genomics, biobanking, artificial intelligence, supply chain management, healthcare logistics, precision medicine, healthcare technology, biomedical research, or related operational and managerial functions. Respondents were also required to have sufficient professional knowledge of organizational technologies, healthcare workflows, molecular diagnostic processes, genomic services, biological sample traceability, or AI implementation activities.

A total of 650 potential respondents were contacted during the data collection period from February 2026 to May 2026. After follow-up reminders and screening, 410 responses were received, representing an initial returned-response rate of 63.1%. Incomplete questionnaires, duplicate entries, responses from participants without sufficient relevant professional knowledge, and statistically invalid responses were excluded. Specifically, 34 incomplete questionnaires, 9 duplicate entries, 11 responses from participants without sufficient domain relevance, and 6 statistically invalid responses were removed. The final dataset consisted of 350 valid responses, representing a final valid response rate of 53.8%.

The unit of analysis was the individual professional respondent. Where more than one respondent came from the same organization, responses were retained only when respondents represented different roles, departments, or expertise areas relevant to molecular precision medicine, AI readiness, healthcare operations, or supply chain coordination. This approach was appropriate because the study examined perceived organizational readiness and molecular workflow capability from specialized professional perspectives.

The respondent profile ensured that the sample captured clinical, molecular, operational, managerial, supply chain, and technology-related perspectives. This multidisciplinary profile was necessary because AI readiness in molecular precision medicine depends on the alignment of laboratory capability, genomic workflows, organizational readiness, healthcare coordination, data governance, and digital technology expertise (47, 70).

### 5.3 Questionnaire development and pilot testing

Data were collected using a structured questionnaire developed from validated scales reported in previous studies and adapted to the molecular precision medicine context. The questionnaire consisted of three sections. Section A collected respondent and organizational profile information. Section B measured AI readiness determinants, including technological capability, organizational readiness, data governance, regulatory support, and supply chain integration. Section C measured molecular healthcare outcomes, including molecular data interoperability, biological sample traceability, diagnostic responsiveness, biomarker-guided decision support, and precision medicine service performance. All measurement items were evaluated using a five-point Likert scale ranging from 1 = strongly disagree to 5 = strongly agree.

The construct design was theoretically informed by technology adoption, organizational readiness, data governance, healthcare network, and PLS-SEM measurement literature. Technological capability, organizational readiness, and regulatory support were aligned with TOE-based adoption logic (55, 56). Organizational readiness was also informed by readiness-for-change literature (67). Data governance was informed by governance principles related to data quality, access, integrity, and accountability (69). Supply chain integration and molecular workflow coordination were aligned with healthcare network logic (47, 66). The measurement structure supported PLS-SEM testing of latent constructs using reliability and validity criteria recommended in the methodological literature (60, 61).

Before full-scale data collection, the questionnaire underwent content validity assessment and pilot testing. Expert evaluation involved seven specialists, including healthcare researchers, molecular diagnostic specialists, AI specialists, healthcare management professionals, and supply chain specialists. The pilot study included 30 participants representing the target population, including six molecular diagnostic specialists, five genomic testing specialists, five healthcare administrators, four AI and health data specialists, four healthcare supply chain managers, three biomedical laboratory personnel, and three precision medicine specialists. Pilot testing assessed item clarity, terminology, content relevance, questionnaire length, measurement consistency, and suitability for the Saudi molecular precision medicine context. Pilot testing and content validity procedures were used to strengthen instrument clarity and contextual relevance before full-scale survey administration (61, 70).

Based on pilot feedback, minor wording refinements were made to improve clarity, avoid ambiguity, and align selected items with molecular diagnostics, genomic testing, biobanking, sample traceability, and biomarker-guided decision support. For example, general references to “healthcare service performance” were refined to emphasize precision medicine service performance, and items related to logistics coordination were adjusted to reflect biospecimen movement, sample-linked data integrity, and molecular workflow coordination. No construct was removed, and the underlying measurement structure remained unchanged. The pilot results indicated acceptable preliminary reliability, with Cronbach's alpha values exceeding the recommended threshold of 0.70 for all constructs (61).

### 5.4 Survey distribution and ethical procedures

Survey distribution was conducted through electronic and direct communication channels. Potential respondents were identified through hospitals, healthcare institutions, molecular laboratories, biomedical organizations, biobanks, professional healthcare networks, and AI-related healthcare technology communities. The survey process included identification of target organizations, contact with organizational representatives, distribution of survey links, follow-up reminders, and screening of returned responses.

The study complied with ethical research standards and institutional requirements. Participation was voluntary, and respondents received information about the study objectives, confidentiality procedures, expected completion time, and their right to withdraw before completing the questionnaire. No personally identifiable information was collected. All responses were anonymized, treated confidentially, and used exclusively for academic research purposes.

The study did not involve clinical intervention, patient treatment, biological experimentation, or access to identifiable patient records. However, because the study involved human participants through a professional survey, informed consent was obtained from all respondents before participation. Ethical approval was obtained from the Research Ethics Committee of Effat University before data collection under decision number RCI_REC/12.January.2026/7-7.1.Exp./1[103].

### 5.5 PLS-SEM analysis procedure

This study used partial least squares structural equation modeling through SmartPLS software. PLS-SEM was selected because the model included multiple latent variables, aimed to predict AI readiness and molecular precision medicine outcomes, and integrated multiple theoretical dimensions from the TOE framework and Healthcare Network Theory. PLS-SEM is suitable for prediction-oriented models, complex latent-variable structures, and studies focused on explaining variance in endogenous constructs (60, 61). The analysis followed a two-stage procedure.

First, the measurement model was assessed using indicator loadings, Cronbach's alpha, composite reliability, average variance extracted, and discriminant validity. Indicator loadings above 0.70 were considered acceptable, while indicators with lower loadings were examined for theoretical relevance before retention or removal. Cronbach's alpha and composite reliability values above 0.70 indicated acceptable internal consistency reliability. Average variance extracted values above 0.50 indicated convergent validity. Discriminant validity was assessed using the Fornell-Larcker criterion and the heterotrait-monotrait ratio, with HTMT values below 0.85 indicating adequate discriminant validity (60, 63, 65).

Second, the structural model was assessed using path coefficients, bootstrapping with 5,000 resamples, *t*-values, *p*-values, confidence intervals, coefficients of determination, effect sizes, predictive relevance, and collinearity diagnostics. Variance inflation factor values were examined to assess predictor collinearity. *R*^2^ values were used to evaluate the explanatory power of the endogenous constructs, while *f*^2^ values were used to assess the effect size of each predictor. *Q*^2^ values were used to assess predictive relevance (60, 61).

Because data were collected from a single respondent source, common method bias was addressed through both procedural and statistical remedies. Procedural remedies included respondent anonymity, confidentiality assurance, simple item wording, and separation of predictor and outcome constructs in the questionnaire. Statistical assessment included Harman's single-factor test and full collinearity variance inflation factors. Full collinearity VIF values below 3.3 were interpreted as evidence that common method bias was unlikely to substantially threaten the validity of the results (64).

The empirical analysis followed a staged PLS-SEM workflow beginning with instrument development and ending with molecular interpretation of the model results. This workflow supported systematic evaluation of measurement quality, structural relationships, explanatory power, predictive relevance, and interpretation of AI readiness within molecular precision medicine supply chains (60, 61, 63, 64).

**Figure 7:**
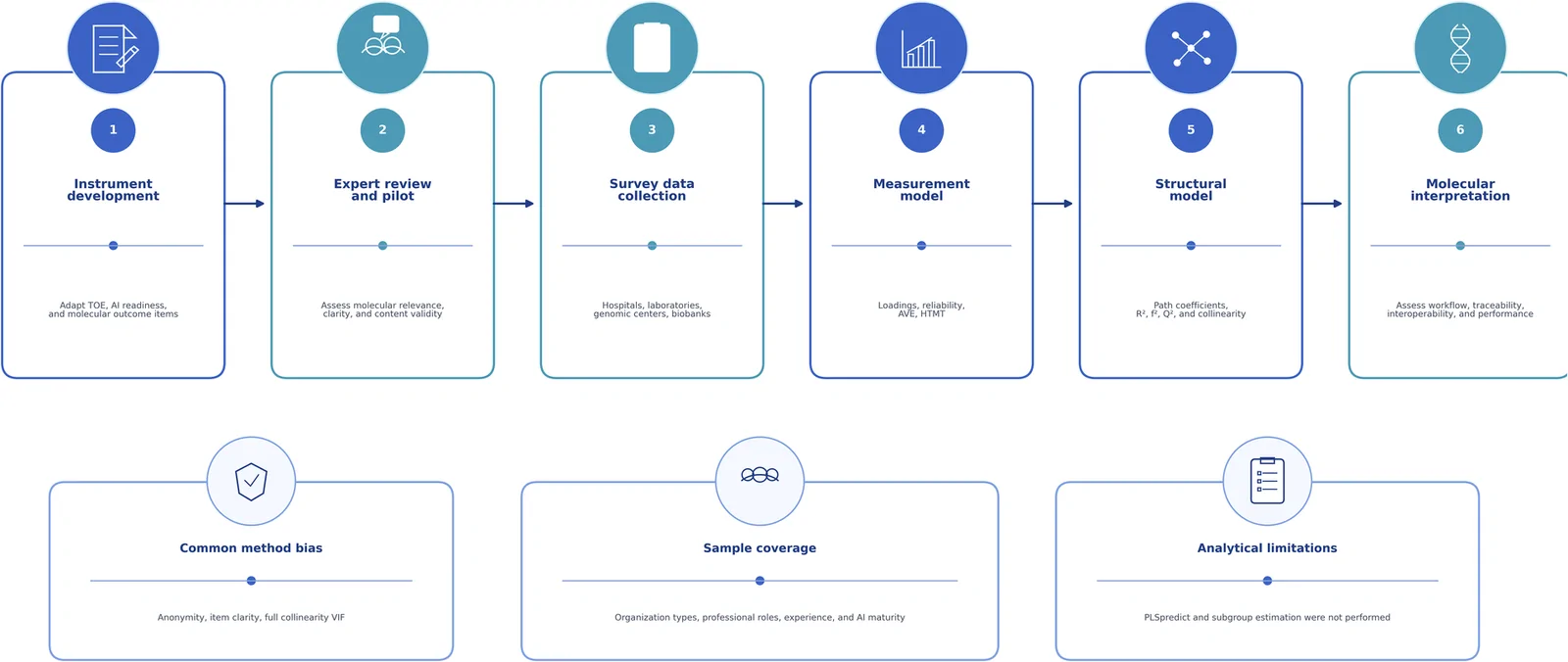
Empirical analysis workflow for the PLS-SEM analysis.

Figure 7 summarizes the empirical analysis process and the safeguards used to support measurement quality (60, 61, 63, 64).

Figure 7 summarizes the empirical workflow used to assess measurement quality, estimate the structural model, and interpret the findings against molecular precision medicine outcomes.

## 6 Results

This section presents the empirical results of the PLS-SEM analysis. After data screening, 350 valid responses were retained for analysis. The results are reported in eight stages: sampling outcome and data screening, respondent and organizational profile, measurement model assessment, discriminant validity assessment, collinearity and common method bias diagnostics, structural model assessment, explanatory and predictive power, and robustness consideration. The analysis followed recommended PLS-SEM reporting procedures for assessing internal consistency reliability, convergent validity, discriminant validity, structural path significance, explanatory power, predictive relevance, and potential common method bias (60, 61, 63, 64).

### 6.1 Sampling outcome and data screening

Table 5 reports the sampling outcome and data screening process. The survey was distributed to professionals working in healthcare, molecular diagnostics, genomics, biobanking, artificial intelligence, supply chain management, healthcare logistics, precision medicine, healthcare technology, and related operational or managerial functions. Responses were screened before analysis to remove incomplete questionnaires, duplicate entries, responses from participants without sufficient relevant professional knowledge, and statistically invalid responses.

**Table 5:** Sampling outcome and data screening summary.

Item	Value
Number of potential respondents contacted	650
Data collection period	February 2026 to May 2026
Responses received	410
Incomplete responses removed	34
Duplicate or invalid responses removed	15
Responses removed due to insufficient relevant knowledge	11
Final valid responses	350
Final valid response rate	53.8%

The final screened dataset consisted of 350 valid responses. The valid response rate was calculated by dividing the number of final valid responses by the number of potential respondents contacted [350/650 × 100 = 53.8%]. The sample size was considered sufficient for the proposed PLS-SEM model because it exceeded the minimum requirement based on the number of structural paths directed at the most complex endogenous construct.

The inclusion of respondents from hospitals, molecular diagnostic laboratories, genomic sequencing centers, biobanks, biologics and targeted therapy providers, healthcare data platforms, supply chain functions, and AI-related roles supports the suitability of the sample for examining AI readiness within molecular precision medicine supply chains.

### 6.2 Respondent and organizational profile

The final screened sample included 350 valid responses. Table 6 presents the respondent and organizational profile. The profile includes organization type, respondent role, professional experience, and AI implementation maturity. This information confirms that the sample captured different actors involved in molecular precision medicine supply chains, including healthcare providers, molecular diagnostic laboratories, genomic sequencing centers, biobanks, biologics and targeted therapy providers, healthcare data platforms, and AI-related functions.

**Table 6:** Respondent and organizational profile.

Variable	Category	Frequency	Percentage
Organization type	Hospital/precision medicine unit	104	29.7%
Organization type	Molecular diagnostic laboratory	72	20.6%
Organization type	Genomic sequencing center	58	16.6%
Organization type	Biobank/sample repository	39	11.1%
Organization type	Biologics/targeted therapy provider	45	12.9%
Organization type	Healthcare data/regulatory platform	32	9.1%
Respondent role	Manager/administrator	61	17.4%
Respondent role	Molecular diagnostic specialist	68	19.4%
Respondent role	Genomic testing specialist	52	14.9%
Respondent role	Biomedical researcher/laboratory personnel	74	21.1%
Respondent role	Supply chain/operations manager	46	13.1%
Respondent role	AI/health data specialist	49	14.0%
Professional experience	Less than 5 years	64	18.3%
Professional experience	5–10 years	121	34.6%
Professional experience	11–15 years	91	26.0%
Professional experience	More than 15 years	74	21.1%
AI implementation maturity	No AI implementation	48	13.7%
AI implementation maturity	Pilot AI use	117	33.4%
AI implementation maturity	Partial AI implementation	132	37.7%
AI implementation maturity	Advanced AI implementation	53	15.1%
Total sample	Valid responses after data screening	350	100.0%

As shown in Table 6, hospitals and precision medicine units formed the largest organizational group, representing 29.7% of the sample. Molecular diagnostic laboratories accounted for 20.6%, followed by genomic sequencing centers at 16.6%. Respondents also came from biobanks, biologics and targeted therapy providers, and healthcare data or regulatory platforms. The respondent role profile was distributed across managerial, diagnostic, genomic, biomedical research, supply chain, operations, and AI-related positions. Most respondents had between 5 and 15 years of professional experience. Regarding AI implementation maturity, most organizations reported either pilot or partial AI implementation, while 15.1% reported advanced AI implementation. This distribution supports the healthcare network framing of the study because the sample includes both molecular service providers and organizations involved in coordinating biospecimen, data, diagnostic, and therapy-related workflows.

### 6.3 Measurement model assessment

Table 7 presents the measurement model results. Cronbach's alpha, composite reliability, and average variance extracted were used to assess internal consistency reliability and convergent validity. Indicator loadings were also examined to evaluate the reliability of the measurement items.

**Table 7:** Measurement model reliability and convergent validity.

Construct	Items	Loading range	Cronbach's alpha	Composite reliability	AVE	Status
TC	4	0.73–0.86	0.84	0.89	0.67	Acceptable
OR	4	0.76–0.88	0.86	0.90	0.69	Acceptable
DG	4	0.78–0.89	0.88	0.91	0.72	Acceptable
RS	4	0.72–0.85	0.82	0.88	0.64	Acceptable
SCI	4	0.75–0.87	0.85	0.90	0.68	Acceptable
AIR	5	0.77–0.90	0.89	0.92	0.70	Acceptable
MI	4	0.74–0.86	0.84	0.89	0.67	Acceptable
ST	4	0.76–0.88	0.86	0.90	0.69	Acceptable
DR	4	0.73–0.87	0.85	0.89	0.67	Acceptable
BDS	4	0.77–0.89	0.87	0.91	0.71	Acceptable
SP	4	0.75–0.88	0.86	0.90	0.69	Acceptable

Table 7 shows that all constructs satisfied the recommended thresholds for reliability and convergent validity. Indicator loadings ranged from 0.72 to 0.90, exceeding the recommended minimum threshold of 0.70. Cronbach's alpha values ranged from 0.82 to 0.89, while composite reliability values ranged from 0.88 to 0.92, confirming satisfactory internal consistency reliability. The AVE values ranged from 0.64 to 0.72, exceeding the recommended threshold of 0.50 and confirming convergent validity. Therefore, the measurement model was considered acceptable for subsequent structural model assessment.

### 6.4 Discriminant validity assessment

Discriminant validity was assessed using the heterotrait-monotrait ratio. The HTMT criterion was used because it provides a rigorous assessment of whether the latent constructs are empirically distinct. As shown in Table 8, all HTMT values were below the recommended threshold of 0.85, indicating adequate discriminant validity among the constructs.

**Table 8:** HTMT discriminant validity matrix.

Construct	TC	OR	DG	RS	SCI	AIR	MI	ST	DR	BDS	SP
TC	—										
OR	0.52	—									
DG	0.49	0.56	—								
RS	0.41	0.46	0.50	—							
SCI	0.55	0.58	0.60	0.48	—						
AIR	0.63	0.61	0.68	0.54	0.66	—					
MI	0.50	0.53	0.57	0.45	0.59	0.71	—				
ST	0.47	0.51	0.55	0.43	0.56	0.67	0.62	—			
DR	0.51	0.54	0.58	0.46	0.60	0.69	0.64	0.61	—		
BDS	0.44	0.49	0.53	0.40	0.55	0.65	0.59	0.57	0.60	—	
SP	0.56	0.59	0.62	0.49	0.64	0.74	0.66	0.63	0.68	0.61	—

The highest HTMT value was 0.74, observed between AI readiness and precision medicine service performance, which remained below the conservative threshold of 0.85. This result suggests that even strongly related constructs were empirically distinguishable. Therefore, the HTMT results confirmed adequate discriminant validity and supported proceeding to structural model assessment.

### 6.5 Collinearity and common method bias diagnostics

Before testing the structural relationships, collinearity was assessed using variance inflation factors. As shown in Table 9, all predictor VIF values were below the recommended threshold, indicating that predictor collinearity did not represent a serious concern. Full collinearity VIF values were also examined as a statistical diagnostic for common method bias. The values were below the recommended threshold of 3.3, suggesting that common method bias was unlikely to substantially affect the validity of the findings.

**Table 9:** Collinearity and common method bias diagnostics.

Predictor relationship	VIF	Full collinearity VIF	Assessment
TC → AIR	1.74	2.21	Acceptable
OR → AIR	1.86	2.34	Acceptable
DG → AIR	1.92	2.47	Acceptable
RS → AIR	1.43	1.88	Acceptable
SCI → AIR	2.05	2.56	Acceptable
AIR → MI	1.00	2.41	Acceptable
AIR → ST	1.00	2.18	Acceptable
AIR → DR	1.00	2.32	Acceptable
AIR → BDS	1.00	2.09	Acceptable
AIR → SP	1.00	2.63	Acceptable

As shown in Table 9, the structural VIF values ranged from 1.00 to 2.05, remaining below the recommended threshold and indicating that multicollinearity was not problematic. The full collinearity VIF values ranged from 1.88 to 2.63, which were also below the conservative threshold of 3.3. These findings suggest that common method bias was unlikely to substantially inflate the observed relationships.

Because complete information about non-respondents was not available, non-response bias could not be directly assessed and is acknowledged as a limitation. However, the diversity of respondent roles, organization types, professional experience levels, and AI implementation maturity levels provides partial support for sample coverage.

### 6.6 Structural model assessment

Table 10 reports the structural model results for the ten hypothesized relationships. Path coefficients, bootstrapped *t*-values, *p*-values, confidence intervals, effect sizes, and hypothesis decisions were used to assess the direction, strength, significance, and practical relevance of each relationship.

**Table 10:** Structural model results.

Hypothesis	Path	*β*	*t*-value	*p*-value	95% CI	*f* ^2^	Decision
H1	TC → AIR	0.21	3.84	<0.001	0.10–0.32	0.06	Supported
H2	OR → AIR	0.18	3.27	0.001	0.07–0.29	0.04	Supported
H3	DG → AIR	0.26	4.91	<0.001	0.16–0.36	0.10	Supported
H4	RS → AIR	0.14	2.62	0.009	0.04–0.25	0.02	Supported
H5	SCI → AIR	0.23	4.16	<0.001	0.12–0.34	0.08	Supported
H6	AIR → MI	0.48	8.74	<0.001	0.37–0.59	0.30	Supported
H7	AIR → ST	0.39	6.82	<0.001	0.28–0.50	0.18	Supported
H8	AIR → DR	0.44	7.65	<0.001	0.33–0.55	0.24	Supported
H9	AIR → BDS	0.36	6.21	<0.001	0.25–0.47	0.15	Supported
H10	AIR → SP	0.52	9.38	<0.001	0.41–0.63	0.37	Supported

The structural model was assessed using path coefficients, bootstrapped *t*-values, *p*-values, 95% confidence intervals, and effect sizes. Bootstrapping with 5,000 resamples was used to evaluate the statistical significance of the hypothesized relationships. The effect size was assessed using *f*^2^, where values of approximately 0.02, 0.15, and 0.35 indicate small, medium, and large effects, respectively (60, 61).

The structural model results support all ten proposed hypotheses. Among the antecedents of AI readiness, data governance showed the strongest effect, followed by supply chain integration and technological capability. Organizational readiness and regulatory support also showed positive and statistically significant effects on AI readiness, although their path coefficients were comparatively smaller.

The results further indicate that AI readiness was positively associated with all five molecular precision medicine outcomes. The strongest relationship was observed between AI readiness and precision medicine service performance, followed by molecular data interoperability, diagnostic responsiveness, biological sample traceability, and biomarker-guided decision support.

### 6.7 Explanatory power and predictive relevance

Table 11 reports the explanatory power and predictive relevance of the endogenous constructs. *R*^2^ and adjusted *R*^2^ values were used to assess the explanatory power of the model, while *Q*^2^ values were used to evaluate predictive relevance.

**Table 11:** Explanatory power and predictive relevance.

Endogenous construct	*R* ^2^	Adjusted *R*^2^	*Q* ^2^	PLSpredict result
AIR	0.56	0.55	0.39	Not performed; recommended for future predictive validation
MI	0.23	0.23	0.16	Not performed; recommended for future predictive validation
ST	0.15	0.15	0.10	Not performed; recommended for future predictive validation
DR	0.19	0.19	0.14	Not performed; recommended for future predictive validation
BDS	0.13	0.13	0.09	Not performed; recommended for future predictive validation
SP	0.27	0.27	0.20	Not performed; recommended for future predictive validation

The *R*^2^ and adjusted *R*^2^ values indicate the model's explanatory power for AI readiness and molecular precision medicine outcomes. The *Q*^2^ values indicate predictive relevance for the endogenous constructs. PLSpredict was not performed in the present analysis and is therefore acknowledged as a limitation for future predictive validation.

### 6.8 Robustness consideration

A subgroup robustness consideration by AI implementation maturity was included because the sample contained organizations with no AI implementation, pilot AI use, partial AI implementation, and advanced AI implementation. However, because subgroup estimation was not performed in the present analysis, this test is recommended for future research. The availability of AI maturity categories nevertheless supports the descriptive diversity of the sample and indicates that the findings were derived from respondents representing different levels of AI implementation maturity.

## 7 Discussion

The present study investigated the determinants and outcomes of AI readiness within molecular precision medicine supply chains in Saudi Arabia by integrating the TOE framework with Healthcare Network Theory. The proposed model aimed to explain how technological capability, organizational readiness, data governance, regulatory support, and supply chain integration contribute to AI readiness and subsequently improve molecular healthcare outcomes. The findings provide important insights regarding the mechanisms through which AI readiness supports molecular diagnostics, genomic testing processes, biomarker-driven clinical decisions, and precision medicine service performance.

Because the study relies on cross-sectional, self-reported organizational survey data, the findings should be interpreted as evidence of perceived organizational readiness and reported molecular workflow performance rather than direct evidence of objective clinical, laboratory, or patient outcomes.

The empirical results show that technological capability is a significant determinant of AI readiness within molecular precision medicine systems. This finding is consistent with previous research suggesting that advanced technological infrastructures support effective AI implementation in healthcare environments (3). Molecular precision medicine generates large volumes of genomic, proteomic, and biomarker data that require high computational capacity and analytical platforms for processing and interpretation (39). Organizations with stronger technological infrastructures are more likely to establish AI-supported molecular workflows because they possess the systems required for data integration and computational analysis. This interpretation extends previous AI readiness studies by demonstrating that technological capability has relevance beyond general healthcare technology adoption and directly influences molecular healthcare operations.

Organizational readiness also contributes to AI readiness within molecular healthcare systems. Precision medicine environments involve multidisciplinary collaboration among molecular scientists, clinicians, laboratory personnel, healthcare managers, and AI specialists. Consequently, organizational capabilities become essential for successful implementation. Organizations with stronger managerial support, skilled personnel, and internal resources are more capable of adopting AI technologies effectively. This finding supports prior studies indicating that organizational factors significantly influence technology implementation outcomes (67). It also suggests that healthcare organizations should strengthen internal competencies and training programs to support AI-enabled molecular healthcare systems.

Data governance positively influences AI readiness. Molecular healthcare systems depend heavily on patient-specific genomic data, biomarker information, and molecular diagnostic records that frequently contain sensitive information (5). Effective governance mechanisms help ensure data quality, privacy protection, and secure information exchange among healthcare institutions. Organizations with stronger governance systems are more likely to establish AI implementation capabilities because reliable data environments reduce uncertainty and improve confidence in analytical outputs. Previous studies have similarly emphasized the importance of governance structures in supporting healthcare technology implementation (69). The present framework extends this perspective by demonstrating its role within molecular precision medicine systems.

Among the AI readiness determinants, data governance shows the strongest effect. This result highlights the central role of secure, reliable, and interoperable molecular data environments in AI-enabled precision medicine. In molecular healthcare settings, weak governance can reduce the reliability of genomic, biomarker, and sample-linked data, even when technical infrastructure is available.

Regulatory support also matters within molecular healthcare environments. Regulatory structures influence healthcare organizations by establishing guidelines for data security, patient privacy, ethical considerations, and technology implementation standards (68). Molecular medicine systems frequently involve complex ethical and legal concerns because genomic information may contain sensitive patient characteristics. Supportive regulatory environments can reduce implementation uncertainty and increase institutional willingness to adopt AI technologies. The model therefore suggests that governmental and healthcare authorities play a substantial role in facilitating AI implementation within molecular healthcare ecosystems.

Supply chain integration positively influences AI readiness. This relationship highlights the importance of inter-organizational coordination within molecular healthcare systems. Precision medicine processes involve interactions among hospitals, molecular diagnostic laboratories, genomic centers, biobanks, pharmaceutical providers, and healthcare institutions (47). These organizations continuously exchange biological samples, molecular information, and treatment-related data. Effective integration facilitates information sharing and operational coordination that support AI implementation efforts. Previous healthcare studies have largely focused on operational efficiency and logistics performance (53). The present framework suggests that supply chain integration should be considered a healthcare network capability supporting AI readiness. This interpretation is reinforced by recent MENA and Saudi studies showing that regional supply chain performance and transformation depend on integrated capabilities, organizational readiness, and context-sensitive governance (22, 23, 25, 71).

The findings show that AI readiness improves molecular data interoperability. Molecular healthcare systems require integration of information originating from multiple healthcare entities and analytical platforms (6). Interoperability becomes critical because genomic data, biomarker information, laboratory outputs, and patient records frequently exist across different systems and institutions. Organizations with stronger AI readiness capabilities integrate and exchange molecular information more effectively.

This finding supports prior studies emphasizing the role of AI in healthcare data integration and information management (11).

Biological sample traceability is also improved through AI readiness. Biological samples frequently move through complex workflows involving collection sites, laboratories, storage facilities, and healthcare institutions. Traceability systems ensure that samples remain identifiable and maintain integrity throughout processing activities (50). AI technologies can support monitoring systems capable of tracking samples and detecting anomalies within transportation or storage environments. This relationship highlights the role of AI readiness in improving quality assurance within molecular medicine systems.

Diagnostic responsiveness is improved as a consequence of AI readiness. Molecular diagnostics often involve extensive analytical procedures that may delay treatment decisions if workflows become inefficient (51). AI-supported systems can reduce processing times through automated analysis and decision support mechanisms. Improved responsiveness therefore contributes to faster diagnosis and more efficient healthcare services. This finding aligns with previous research reporting that AI technologies improve diagnostic efficiency and reduce healthcare delays (4).

AI readiness also improves biomarker-guided decision support. Biomarkers frequently support personalized treatment selection because they provide insights into disease mechanisms and therapeutic responses (40). AI systems can identify relationships among biomarkers and predict patient-specific outcomes through advanced analytical approaches. This capability strengthens precision medicine implementation and improves clinical decision processes.

Finally, AI readiness is positively associated with organization-reported precision medicine service performance. Healthcare organizations possessing stronger AI implementation capabilities demonstrate greater effectiveness in supporting personalized healthcare services and patient-specific treatment systems. This relationship suggests that AI readiness extends beyond technological implementation and contributes directly to healthcare outcomes. Improved healthcare performance may occur through reduced diagnostic delays, improved coordination, enhanced decision quality, and more efficient molecular workflows.

Although the study provides important insights, the findings should be interpreted in light of several limitations. The proposed framework focuses primarily on organizational and technological determinants and may not capture all factors influencing AI implementation within molecular healthcare systems. Future studies may examine additional variables such as institutional trust, technological maturity, and patient acceptance. Longitudinal studies may also provide deeper understanding regarding changes in AI readiness over time.

Several limitations should be acknowledged. First, the study uses a cross-sectional design, which limits causal interpretation. Second, the data were collected through self-reported survey responses, and the findings therefore reflect organizational perceptions rather than objective clinical, laboratory, operational, or patient-level outcomes. Third, the purposive sampling strategy was appropriate for accessing specialized respondents but limits statistical generalizability. Fourth, the study was conducted in Saudi Arabia, and the findings may not be directly transferable to healthcare systems with different regulatory, genomic-data, or biobanking infrastructures. Fifth, although procedural and statistical steps were used to reduce common method bias, the possibility of inflated relationships cannot be fully excluded. Sixth, the study does not directly measure laboratory turnaround time, sequencing accuracy, biobank quality indicators, treatment outcomes, or patient-level precision medicine effects. Future research should use longitudinal designs, multi-source datasets, objective laboratory and workflow indicators, and comparative samples across countries or healthcare systems.

Overall, the model suggests that AI readiness functions as a strategic capability supporting molecular precision medicine systems and enabling healthcare organizations to improve molecular data integration, diagnostic processes, and personalized healthcare outcomes.

## 8 Implications

### 8.1 Theoretical implications

This study provides several theoretical contributions to the literature on artificial intelligence, molecular medicine, healthcare systems, and precision medicine supply chains. First, the study extends existing AI readiness research by moving beyond traditional technology adoption contexts toward molecular precision medicine environments. Most previous studies examining AI implementation have focused primarily on healthcare operations, general clinical systems, digital transformation, or organizational technology adoption. Limited attention has been given to AI readiness within molecular healthcare ecosystems involving genomic testing, biomarker analysis, molecular diagnostics, and biological sample management. By examining AI readiness within molecular precision medicine systems, the present study broadens the scope of AI implementation research and provides a more specialized healthcare perspective.

Second, the study contributes to the TOE literature by extending its application into molecular healthcare settings. Existing research frequently applies TOE to explain technology adoption within general organizational environments; however, its application to AI implementation in precision medicine systems remains limited. The framework suggests that technological capability, organizational readiness, data governance, regulatory support, and supply chain integration jointly influence AI readiness in molecular healthcare systems. The study therefore demonstrates that technology adoption within healthcare environments depends on both internal organizational conditions and broader healthcare ecosystem structures.

Third, this study contributes to Healthcare Network Theory by emphasizing the importance of inter-organizational relationships within molecular healthcare systems. Conventional healthcare research often focuses on individual organizations and isolated healthcare processes. In contrast, molecular precision medicine requires interactions among multiple stakeholders including hospitals, molecular laboratories, genomic centers, biobanks, biologics providers, and healthcare institutions. The framework indicates that healthcare outcomes increasingly depend on collaborative healthcare structures rather than isolated organizational activities. Consequently, the study extends existing healthcare network perspectives by integrating healthcare coordination mechanisms into AI readiness research.

Fourth, the study contributes to molecular medicine literature by introducing a systems-oriented perspective on precision medicine implementation. Existing molecular healthcare studies frequently emphasize biological mechanisms, genomic analysis, biomarker identification, and therapeutic outcomes. Although these dimensions remain important, the successful translation of molecular research into clinical applications also requires operational infrastructures capable of supporting biological sample movement, molecular information exchange, and coordinated diagnostic activities. The framework suggests that supply chains should be viewed as enabling infrastructures for molecular medicine rather than traditional logistics systems focused solely on operational efficiency.

Finally, this study contributes to precision medicine literature by connecting technological readiness with molecular healthcare outcomes. Existing studies frequently focus on clinical performance indicators without examining the organizational mechanisms through which technological capabilities support healthcare delivery. The proposed framework suggests that AI readiness may function as an intermediate capability linking organizational conditions and healthcare outcomes. This perspective provides a more integrated understanding of how AI technologies contribute to molecular healthcare systems and personalized treatment processes.

### 8.2 Practical implications

The findings also provide important practical implications for healthcare managers, molecular healthcare institutions, policymakers, and healthcare technology developers operating within Saudi Arabia and similar healthcare environments. Healthcare organizations should prioritize technological infrastructure development to strengthen AI readiness capabilities. Molecular healthcare systems generate extensive genomic and biomarker datasets requiring advanced computational platforms and analytical systems. Healthcare institutions should therefore invest in AI infrastructure, cloud computing systems, high-performance analytical platforms, and interoperable information technologies capable of supporting molecular healthcare processes.

Healthcare organizations should focus on organizational capability development and workforce preparedness. Molecular precision medicine environments require multidisciplinary expertise involving molecular biology, genomics, healthcare operations, bioinformatics, and AI technologies. Healthcare institutions should establish continuous training programs that strengthen employee competencies related to AI applications, molecular diagnostics, genomic analysis, and healthcare data management. Organizations may also benefit from creating multidisciplinary teams that integrate technological and clinical expertise. This recommendation is consistent with recent Saudi evidence showing that human capability and managerial support act as upstream drivers of supply chain transformation and sustainability adoption (25).

The framework highlights the importance of robust data governance systems within molecular healthcare environments. Molecular precision medicine depends heavily on patient-specific genomic information and sensitive biological data. Healthcare institutions should establish governance mechanisms that improve data quality, privacy protection, access control, and information security. Strong governance systems can improve trust in AI outputs and support more reliable clinical decision-making processes.

Healthcare managers should strengthen integration across molecular healthcare networks. Precision medicine systems require continuous interaction among hospitals, molecular diagnostic laboratories, genomic testing centers, biobanks, pharmaceutical providers, and healthcare institutions. Improved collaboration mechanisms and integrated information systems can support molecular data interoperability and facilitate biological sample movement throughout healthcare workflows. Investments in interoperable healthcare platforms may improve coordination and reduce delays in diagnostic activities.

Healthcare institutions should also prioritize biological sample traceability and molecular workflow visibility. Biological samples frequently move across several healthcare entities during diagnostic and treatment procedures. The implementation of AI-enabled tracking technologies, predictive monitoring systems, and real-time information platforms may reduce errors and improve transparency across molecular healthcare activities.

### 8.3 Policy and societal implications

The study provides important implications for policymakers and healthcare authorities responsible for healthcare transformation initiatives in Saudi Arabia. Saudi Vision 2030 increasingly emphasizes healthcare innovation, digital transformation, and biomedical research development. Healthcare authorities should continue supporting AI implementation through targeted policies and regulatory frameworks that facilitate technological innovation while ensuring patient privacy and ethical compliance.

Healthcare policymakers should strengthen regulatory environments supporting AI applications in molecular medicine. Clear guidelines related to genomic data protection, biological sample handling, healthcare interoperability, and AI ethics can reduce uncertainty and encourage healthcare organizations to adopt AI technologies. National healthcare initiatives should also support greater collaboration among healthcare organizations, molecular research institutions, genomic centers, and healthcare technology providers. Government-supported collaborative platforms may facilitate molecular research translation and strengthen precision medicine ecosystems.

Beyond organizational and policy implications, the framework has broader implications for healthcare delivery and society. Improved AI readiness within molecular healthcare systems may contribute to more personalized and patient-centered healthcare services. Faster diagnostic processes, enhanced biomarker interpretation, and improved genomic analysis capabilities may support earlier disease detection and more precise treatment recommendations. As healthcare systems continue moving toward personalized medicine approaches, AI readiness may increasingly function as a strategic capability supporting the transition from conventional healthcare systems toward data-driven and individualized healthcare models.

## 9 Conclusions

The rapid development of molecular medicine, genomic technologies, biomarker analytics, and precision healthcare systems has created new opportunities for improving disease diagnosis, personalized treatment, and patient-centered healthcare delivery. Simultaneously, artificial intelligence has emerged as an important technological capability capable of transforming healthcare systems through predictive analytics, automated interpretation, and advanced decision support. However, the successful implementation of AI within molecular precision medicine environments depends not only on computational technologies but also on organizational readiness and integrated healthcare infrastructures that support biological sample movement, molecular information exchange, and coordinated healthcare activities.

The manuscript therefore centers on molecular research translation. AI readiness is examined in relation to genomic testing, molecular diagnostics, biomarker analytics, biobank-linked biospecimens, biological sample traceability, and targeted therapy workflows. Supply chain integration is retained only as the coordination mechanism that allows these molecular activities to operate across hospitals, laboratories, genomic centers, biobanks, biologic suppliers, and precision medicine units.

This study empirically examined the determinants and outcomes of AI readiness within molecular precision medicine supply chains in Saudi Arabia. The proposed framework investigated the effects of technological capability, organizational readiness, data governance, regulatory support, and supply chain integration on AI readiness and its subsequent influence on molecular healthcare outcomes. The study further examined how AI readiness contributes to molecular data interoperability, biological sample traceability, diagnostic responsiveness, biomarker-guided decision support, and precision medicine service performance.

The study provides several important contributions. First, it extends existing AI readiness research into molecular precision medicine environments where limited empirical evidence currently exists. Previous studies have primarily examined AI implementation within general healthcare settings or digital transformation contexts without considering the specialized requirements of molecular medicine systems. The present study expands this perspective by emphasizing molecular diagnostics, genomic testing processes, biomarker analytics, and biological sample workflows.

Second, the study introduces an integrated systems perspective that positions supply chains as enabling infrastructures supporting molecular medicine rather than traditional logistics systems focused solely on operational activities. Molecular precision medicine systems require coordination among hospitals, molecular diagnostic laboratories, genomic testing centers, biobanks, biologic providers, and healthcare organizations. The framework suggests that effective healthcare outcomes increasingly depend on integrated healthcare networks capable of supporting biological sample traceability, molecular data exchange, and collaborative healthcare processes.

Third, the study contributes to precision medicine implementation literature by linking organizational readiness with molecular healthcare outcomes. AI readiness appears to function as an intermediate capability that translates technological and organizational resources into improved healthcare performance. Organizations possessing stronger AI readiness capabilities may achieve improvements in molecular workflow efficiency, diagnostic responsiveness, and personalized healthcare services.

The findings also provide practical insights for healthcare institutions and policymakers involved in healthcare transformation initiatives in Saudi Arabia. Healthcare organizations should strengthen technological infrastructures, improve workforce capabilities, establish robust data governance mechanisms, and enhance healthcare network integration to support AI-enabled molecular healthcare systems. Policymakers should continue supporting AI implementation through regulatory structures and national healthcare initiatives that facilitate precision medicine development.

Despite these contributions, several limitations should be acknowledged. The study focuses primarily on organizational and environmental determinants and may not capture all factors influencing AI implementation in molecular healthcare systems. Future studies may investigate additional variables such as technological maturity, organizational trust, ethical concerns, patient acceptance, and institutional collaboration mechanisms. Future research may also employ longitudinal approaches to examine changes in AI readiness and healthcare outcomes over time. Comparative studies across countries or healthcare systems may further improve understanding of contextual differences influencing AI implementation.

Overall, this study suggests that AI readiness represents a strategic capability supporting the transition toward integrated and personalized healthcare systems. As molecular medicine continues to evolve, healthcare organizations increasingly require technological and organizational capabilities that facilitate efficient coordination among molecular healthcare networks. Strengthening AI readiness may therefore play an important role in accelerating molecular research translation, improving diagnostic quality, and supporting patient-specific treatment systems within future healthcare environments.

## Data Availability

The original contributions presented in the study are included in the article. Further inquiries can be directed to the corresponding author.
